# Case report: Adjuvant therapy with toceranib for an incompletely resected renal cell carcinoma with suspected pulmonary metastasis in a dog

**DOI:** 10.3389/fvets.2023.1287185

**Published:** 2023-11-13

**Authors:** Da-Eun Lee, Chang-Hoon Nam, Hun-Young Yoon, Kieun Bae, Kyong-Ah Yoon, Jung-Hyun Kim

**Affiliations:** ^1^Department of Veterinary Internal Medicine, College of Veterinary Medicine, Konkuk University, Seoul, Republic of Korea; ^2^Department of Veterinary Surgery, College of Veterinary Medicine, Konkuk University, Seoul, Republic of Korea; ^3^Department of Biochemistry, College of Veterinary Medicine, Konkuk University, Seoul, Republic of Korea

**Keywords:** adjuvant chemotherapy, canine renal cell carcinoma, pulmonic metastasis, tyrosine kinase inhibitor, toceranib phosphate

## Abstract

Primary renal neoplasia is rare in humans and dogs, with renal cell carcinoma (RCC) being the most common form of this cancer. As RCC is often diagnosed at an advanced stage, pulmonary metastasis is frequently observed. Tyrosine kinase inhibitors (TKIs) are the standard adjuvant treatments for metastatic RCC in humans. Similarly, in veterinary medicine, recent trials have employed TKIs for early-stage RCC patients who underwent complete surgical resection and showed no distant metastasis. However, the use of TKIs has not yet been reported commonly in cases of advanced RCC with metastasis. This case study presents the first clinical outcomes of TKI therapy in a dog with incompletely resected RCC and metastasis. A 5-year-old spayed female Chihuahua was referred to our hospital with a right renal mass and multiple pulmonary nodules suspected to be metastases. A portion of the renal mass was surgically removed, and histopathological examination revealed RCC with a high mitotic index. Adjuvant chemotherapy was administered, owing to incomplete resection with suspected pulmonary metastasis. An anticancer drug response prediction test was conducted using patient tissues. Since toceranib showed the most favorable responsiveness, it was selected as a therapeutic agent. Toceranib was orally administered at a dosage of 2.27 mg/kg every 48 h. Regular medical records for potential adverse effects were obtained, including systemic blood pressure, complete blood count, serum biochemical examination, and urinalysis. After 2 weeks of toceranib therapy, partial remission of pulmonary nodules continued for 2 months. The patient did not experience any adverse effects of the anticancer drug during the 4-month follow-up period. However, the patient died from an unidentified cause 6 months after the initial detection of the renal mass. This report describes the use of toceranib in dogs with RCC. In the present case, the patient showed an initial response to chemotherapy, and despite the presence of several poor prognostic factors, the dog survived beyond the expected 3-month lifespan to 6 months. Notably, no adverse events were observed during treatment.

## 1. Introduction

Primary renal neoplasia is not commonly found in dogs, with a prevalence of 0.3–1.5% among all canine primary neoplasms (1). Renal cell carcinoma (RCC) constitutes approximately 49–65% of all primary renal neoplasms (2). The commonly reported signs of renal neoplasia in dogs include weight loss, lethargy, hematuria, polydipsia, polyuria, and hematuria (3). RCC is often identified at an advanced stage of the disease, with 18–48% of cases showing radiographic findings suggestive of pulmonary metastasis on thoracic imaging (1). Nephrectomy is considered the primary treatment option for RCC in dogs and has been reported to result in longer survival periods compared to cases where only medical therapy is administered (3).

Tyrosine kinase inhibitors (TKIs) are small molecules that bind to receptor tyrosine kinases (RTKs) and inhibit the phosphorylation of downstream target proteins (4). In veterinary medicine, TKIs have been developed as therapeutic options for mast cell tumors that cannot be removed surgically in dogs (4). Toceranib phosphate, a TKI, is widely employed in veterinary oncology and has been approved as a targeted therapeutic agent by the Food and Drug Administration (5). Compared with surgery alone, the use of toceranib phosphate as adjuvant chemotherapy for adenocarcinoma, including RCC, in dogs has been shown to increase the median time to progression compared with surgery alone (6). In human medicine, TKIs are the standard treatment for metastatic RCC (mRCC), and their effectiveness has been demonstrated (7–9). Therapy for mRCC involves targeting the vascular endothelial growth factor and the mammalian target of rapamycin pathways (10). In the present case, a dog with an incompletely resected RCC and suspected pulmonary metastasis underwent nephrectomy followed by adjuvant chemotherapy with toceranib. This report discusses the clinical presentation, diagnostic process, treatment approach, and applications of toceranib in the management of RCC in a canine.

## 2. Case description

A 5-year-old spayed female Chihuahua, weighing 2.2 kg, was referred to our institution for evaluation of a right renal mass and multiple pulmonary nodules detected during a medical check-up for anorexia at a local animal hospital. The owner reported that the patient showed sudden decrease in appetite and energy level. The patient also exhibited occasional open mouth breathing and increased respiratory rate. Physical examination revealed normal vital signs, including body temperature, pulse rate, respiratory rate, and blood pressure.

Complete blood count (CBC) showed no remarkable findings. The serum biochemical profile showed hyperglobulinemia (4.6 g/dL; reference range, 2.5–4.5 g/dL), elevated aspartate aminotransferase (99 U/L; reference range, 0–50 U/L) and gamma-glutamyl transferase (37 U/L; reference range, 0–7 U/L) as well as hyperlactatemia (6.23 mmol/L; reference range, 0.5–2.5 mmol/L), and elevated C-reactive protein (7.9 mg/dL; reference range, 0.1–1 mg/dL).

**Figure 1:**
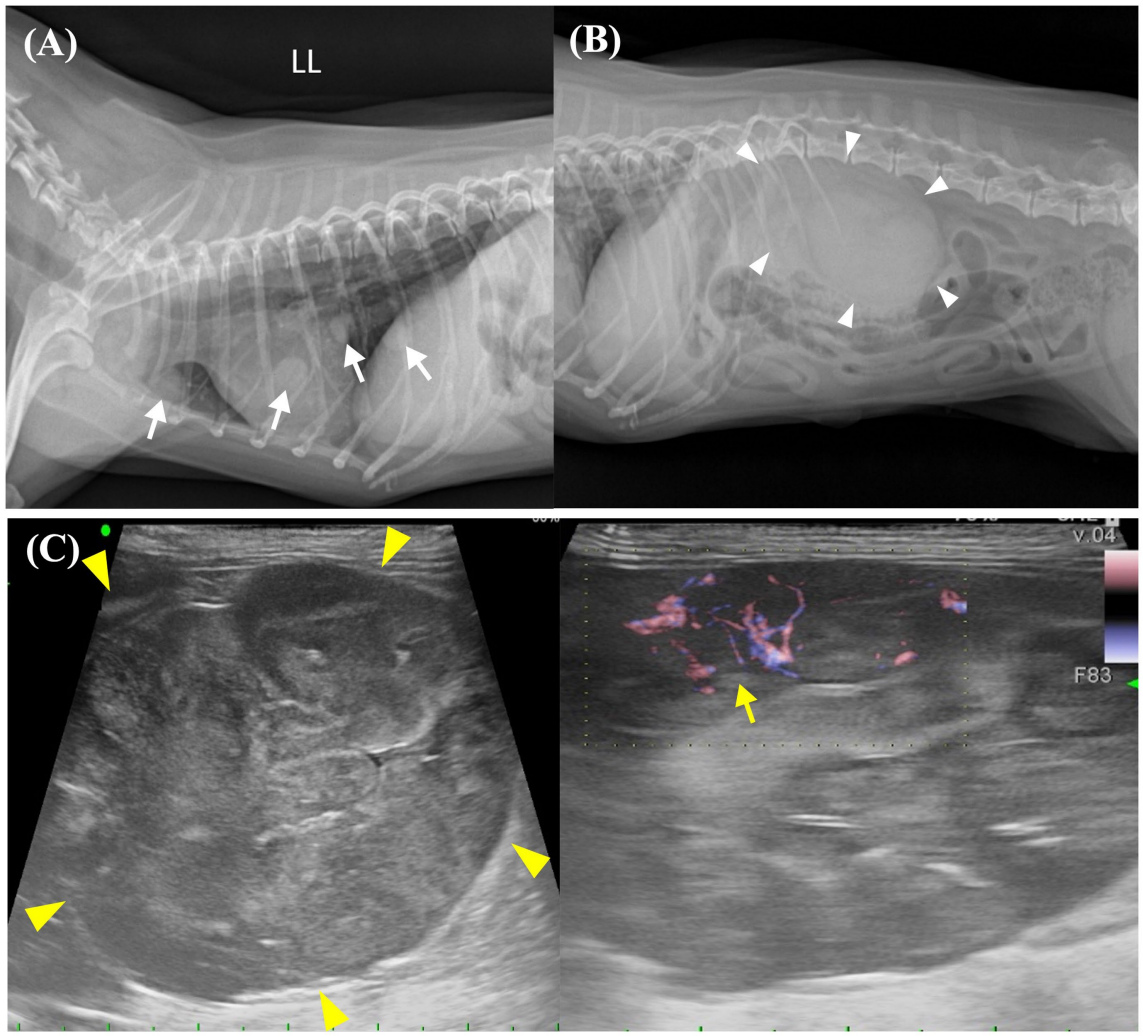
Preoperative imaging of the pulmonary nodules and abdominal mass. **(A)** Thoracic radiographic image shows the presence of numerous pulmonary nodules with diameters ranging from 5 to 11 mm, which are distributed extensively throughout the lung field. Identifiable pulmonary nodules are indicated by white arrows. **(B)** Abdominal radiographic image shows a soft tissue opacity in the region of the right kidney (white arrowheads). **(C)** Abdominal ultrasound image shows a large (53.8 × 37.0 mm), heterogenous mass (yellow arrowheads) arising from the cranial pole of the right kidney. The right kidney is accompanied by an active vascular response on E-flow (yellow arrow). LL, left lateral view.

**Figure 2:**
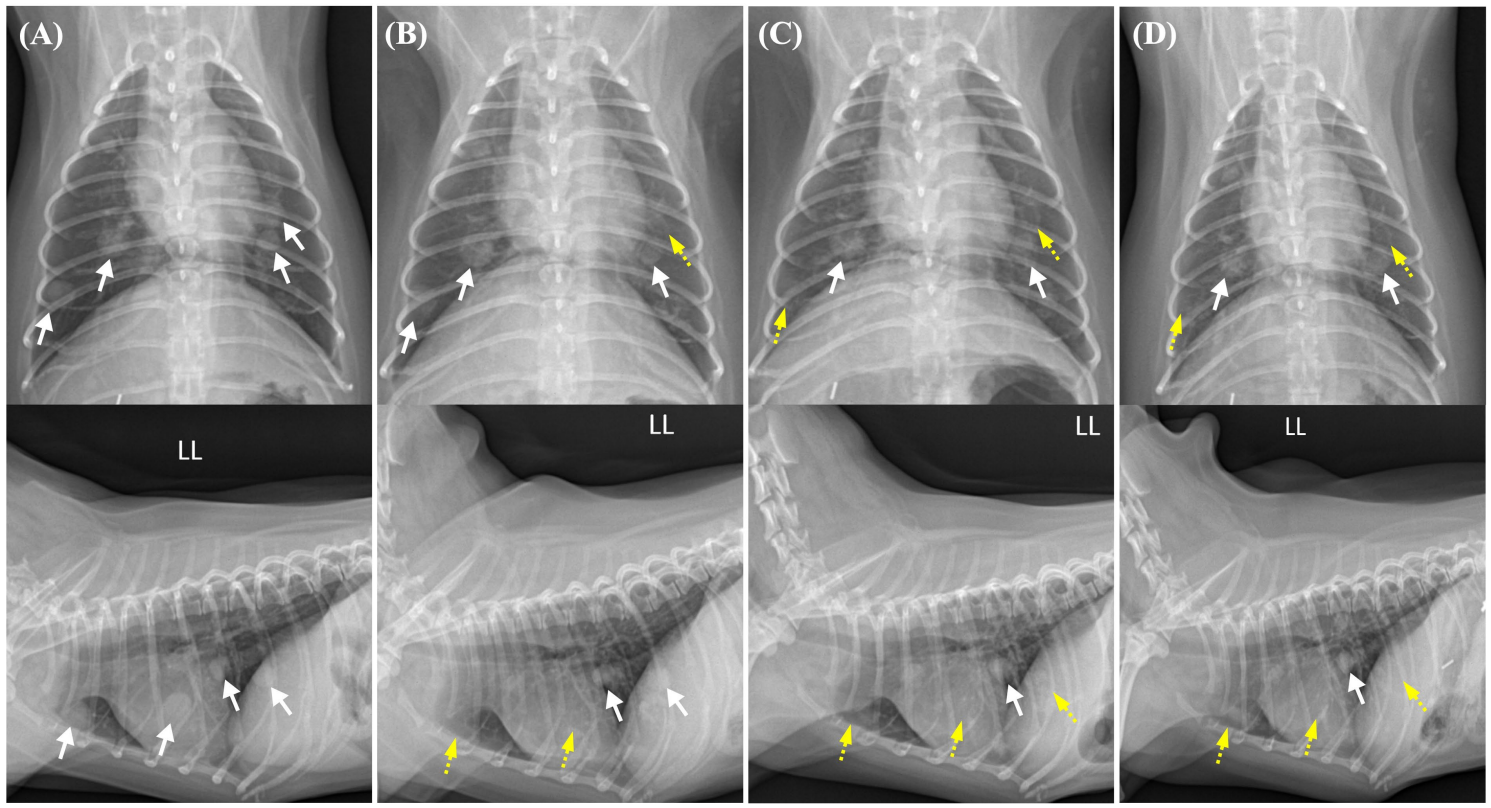
Changes in the pulmonary nodules on thoracic radiographs from before toceranib administration to 2 weeks, 2 months, and 4 months after treatment initiation. Pulmonary nodules with well-defined borders are indicated by white arrows, whereas regressed nodules are indicated by yellow dashed arrows. **(A)** Prior to toceranib administration, multiple pulmonary nodules with soft tissue opacity are observed throughout the generalized lung field. The pulmonary nodule with the largest size measures 11.4 × 8.8 mm. **(B)** In a thoracic radiograph obtained 2 weeks after toceranib administration, the previously identified pulmonary nodule with the largest size is no longer visible in the lateral view. The largest measurable diameter of the remaining nodule is approximately 9 mm. **(C)** In a thoracic radiograph obtained 2 months after toceranib administration, another pulmonary nodule initially sized 8.4 × 7.7 mm is no longer detected. The majority of the pulmonary nodules exhibit partial remission and a diminished size. **(D)** In a thoracic radiograph obtained 4 months after toceranib administration, no further remission is observed in the existing pulmonary nodules. Indications of newly detected nodules or the recurrence of previously diminished pulmonary nodules are also not found.

Thoracic radiography images showed multiple pulmonary nodules of different sizes ranging from 5 to 11 mm, with a maximum size of 11.4 × 8.8 mm (Figures 1A, 2A). Abdominal radiography revealed a soft tissue opacity between the 13th thoracic vertebra (T13) and the third lumbar vertebra (L3) in the lateral view (Figure 1B). Additionally, abdominal ultrasound revealed a heterogeneous mass, 53.8 × 37.9 mm in size, arising from the cranial pole of the right kidney with a vigorous blood flow response on E-flow (Figure 1C). A portion of the caudal aspect of the right kidney retained its normal structure, whereas the remainder underwent tumor transformation. The other organs and adjacent lymph nodes were unremarkable.

**Figure 3:**
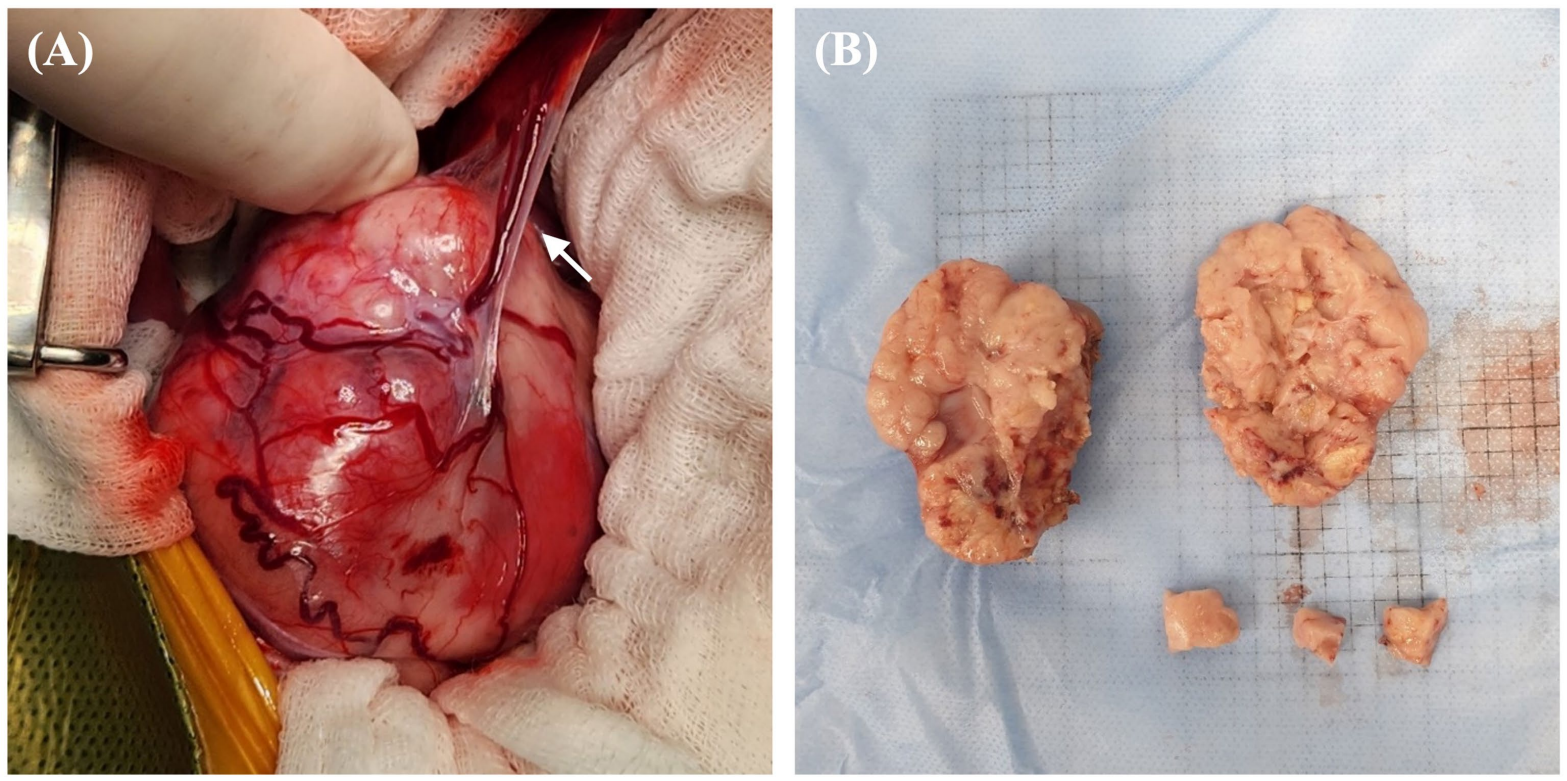
Gross morphological features of right renal mass in a dog with RCC. **(A)** Intraoperative finding of a highly vascularized renal mass with blood vessels connecting to the liver and potentially functioning as feeding vessels (arrow). Numerous tortuous vessels are found to have surrounded the capsule of the mass. **(B)** Surgically removed mass of size 50 × 65 mm, with a lumpy bumpy parenchyma, an indistinct corticomedullary junction, and an irregular margin; findings suggest a fully tumorous transformation with total loss of normal structure. RCC, renal cell carcinoma.

**Figure 4:**
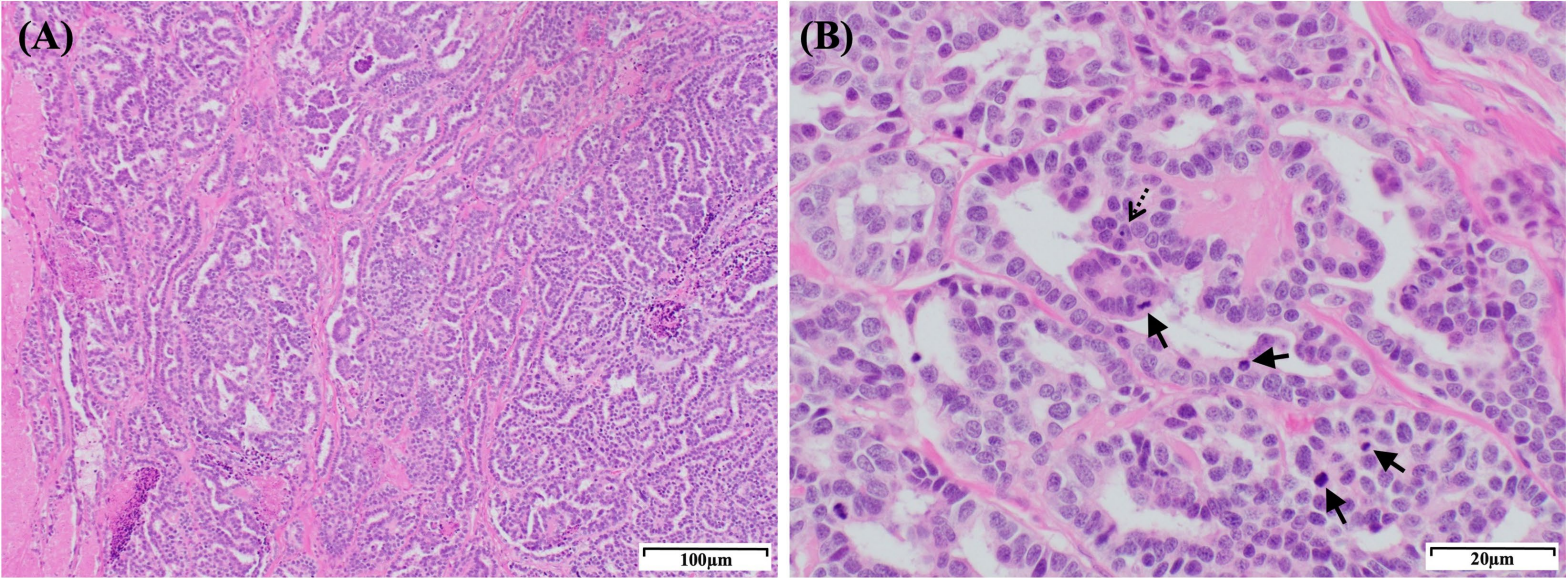
Histopathological features of the right renal mass in a dog with a papillary RCC. **(A)** Histological section showing a papillary RCC. The tumor consists of densely packed, well-defined, and non-encapsulated neoplastic polygonal cells arranged in a palisading pattern. The neoplastic cells form ducts, papillary projections, and lobules on a fine fibrovascular stroma. H&E staining: magnification, 100×; scale bar, 100 μm. **(B)** The tumor cells exhibit dense chromatin and 1–4 prominent nucleoli (dashed arrow) with variably distinct cell borders, moderate-to-scant eosinophilic cytoplasm, and regional vacuolization. Moderate anisocytosis and anisokaryosis are observed with a high number of mitotic figures (arrows; 66 in 10 high-power fields). H&E staining: magnification, 400×; scale bar, 20 μm. RCC, renal cell carcinoma; H&E, hematoxylin & eosin.

The patient was deemed unsuitable for a computed tomography examination due to its condition and the risk posed by anesthesia. Thus, we proceeded to perform right nephrectomy. Intraoperatively, the renal mass was observed to have adhered extensively to the caudal vena cava, indicating significant involvement of the tumor in the vessel. Owing to this extensive adhesion, incomplete resection was performed without including the tumor within the caudal vena cava. Tumor invasion into the ipsilateral adrenal gland was grossly identified intraoperatively; therefore, the adrenal gland was resected. Additionally, blood vessels connecting the tumor to the liver were identified and suspected to function as feeding vessels (Figure 3A). The excised tissue samples were subjected to histopathologic evaluation (Figure 3B), which confirmed that the renal mass represented papillary RCC with incomplete excision margins (Figure 4A). Considering that a tumor, node, and metastasis (TNM) staging system has not been established for RCC in dogs, the patient’s TNM stage was determined to be T4N0M1 using the human TNM staging system (11). Histopathological examination revealed high malignancy with dense chromatin, prominent nucleoli, and numerous mitotic figures (66 in 10 high-power fields; Figure 4B). Approximately 45–55% of the mass comprised multifocal tumor necrosis invading the surrounding parenchyma, characterized by pyknotic cells and nuclear fading. The surrounding renal parenchyma exhibited compression with moderate interstitial clear spacing, mild tubular dilation, and multifocal lymphocyte aggregation.

To determine the most effective anticancer drug for this patient, we performed an *in vitro* ATP-based anticancer drug response prediction test using a cell viability assay. Following surgery, the patient’s tumor sample was harvested and lysed using collagenase II (Life Technologies, Carlsbad, CA, United States), hyaluronidase, and Ly27632 (Sigma-Aldrich, St. Louis, MO, United States) at 37°C. After filtration through a 70-μm cell strainer (BD Biosciences, San Diego, CA, United States), the tumor sample was dissociated into single cells. These cells were cultured in Advanced Dulbecco’s modified Eagle’s medium/F12 supplemented with 10% fetal bovine serum, 10 mM N-2-hydroxyethylpiperazine-N′-2-ethanesulfonic acid, along with GlutaMAX^™^ (Thermo Fisher Scientific, Waltham, MA, United States), and Zellshield^®^ (Minerva Biolabs, Berlin, Germany). Subsequently, the cells (1 × 10^4^) were seeded in 96-well plates and incubated overnight at 37°C in a 5% CO_2_ humidified atmosphere. The medium was replaced with a gradient of chemotherapy drug concentrations. After 24 h, cell viability was assessed using the CellTiter-Glo^®^ Luminescent Cell Viability Assay (Promega, Madison, WI, United States), following the manufacturer’s instructions. The results demonstrated that among sorafenib, imatinib, doxorubicin, carboplatin, and toceranib (Palladia^®^, Zoetis, Florham Park, NJ, United States), toceranib showed the most potent cytotoxic effect (Supplementary Figure S1). In contrast to carboplatin, which did not induce cell death even at a high concentration of 100 μM, toceranib displayed stronger antitumor efficacy, even at the lowest concentration of 6 μM, compared to other chemotherapeutic agents.

Thus, 1 month after the surgery, toceranib administration was initiated at 2.27 mg/kg orally every 48 h, in accordance with the label’s minimum dosage recommendation. The dose was adjusted with consideration for possible adverse effects and potential future dose escalation. The medical recheck intervals for adverse drug events, tumor recurrence, and metastasis were once per week for the first 2 weeks, followed by every 2 weeks for 4 weeks and monthly thereafter. At the 2-week follow-up examination, no adverse effects were observed following assessments of systemic blood pressure measurement, CBC, serum chemistry profile, coagulation profile, and urinalysis, based on the veterinary cooperative oncology group-common terminology criteria for adverse events (VCOG-CTCAE) criteria (12). Serial changes in the pulmonary nodules following the administration of toceranib are shown in Figure 2. Pulmonary nodules, suspected to be metastases of renal cell carcinoma, exhibited a decrease in size and opacity on thoracic radiography, accompanied by blurring of their borders (Figure 2B). The owner reported that the dog was active and exhibited normal eating, drinking, urination, and stable breathing.

At the 2-month check-up, the patient was observed to have consistently maintained a good condition. The owner reported that the patient appeared to be pain-free, displaying high energy levels and a strong appetite. No remarkable findings were observed concerning the tumors or adverse effects of toceranib. On thoracic radiography, most of the pulmonary nodules exhibited partial remission and had diminished in size (Figure 2C). Abdominal ultrasonography revealed no additional findings suggestive of RCC metastasis.

At the 4-month check-up, in comparison to the thoracic radiograph from 2 months ago, there was no further regression observed in the existing pulmonary nodules; they appeared similar. Nevertheless, there were no signs of recurrence in those that had previously shown remission, and no additional nodules were detected (Figure 2D). Similarly, no adverse effects related to chemotherapy were observed according to the VCOG-CTCAE criteria (12). However, 1 week before the scheduled 5-month check-up, the patient experienced severe fatigue, vomiting, and diarrhea and died at home without a definitive cause. This occurred 6 months after the initial detection of the renal mass and subsequent surgery and 5 months after the administration of toceranib. A necropsy was not performed because of the owner’s refusal.

## 3. Discussion

RCC exhibits a low response to chemotherapy, and nephrectomy is recognized as the most effective treatment option for dogs (3). Surgery contributes to prolonging the survival time in dogs with RCC. However, the role of adjuvant chemotherapy in prolonging survival time has not been fully established (3). Although adjuvant chemotherapy, including administration of conventional chemotherapeutic agents, TKIs, and metronomic chemotherapy, following surgery has been reported, there has been no significant extension of the median survival time compared to that in cases where adjuvant chemotherapy has not been administered (2). Only one study has reported an extended median survival time in dogs with RCC who received toceranib as adjuvant chemotherapy after surgery compared to those who underwent surgery alone (6).

The mitotic index (MI) is a significant prognostic indicator for dogs with various types of cancers, including mast cell tumors, melanomas, mammary carcinomas, and soft tissue sarcomas (2). Additionally, MI is strongly associated with the tumor grade of certain canine mast cell tumors (13). It has also been identified as a reliable prognostic factor in dogs with RCC, with reported survival durations of 40 months in cases with MI < 10, and 15 months in cases with MI ranging from 10 to 30 (1). The median survival has been reported to be 4–6 months with MI >30 (1, 2). In the present case, histopathological examination revealed an MI of 66, indicating a significant degree of malignancy.

Carvalho et al. observed a significantly shorter median survival time in dogs with mRCC than in those without metastasis at the time of diagnosis (2). Additionally, dogs with completely resected RCC without metastasis have been reported to have a survival period of 3–4 months without adjuvant chemotherapy (14, 15). However, no data are available on the survival period of dogs with mRCC who have undergone incomplete renal mass resection. Therefore, we anticipated that our patient’s prognosis would not be favorable, even with chemotherapy, and estimated the survival time as less than 3 months after considering the incomplete resection performed, presence of suspected pulmonary metastasis, and the high MI. However, the patient survived for up to 6 months from the time of the initial detection of the renal mass.

For the anticancer drug response prediction test, we chose sorafenib, imatinib, doxorubicin, carboplatin, and toceranib based on their previous application history in humans and dogs. Among these five conventional and targeted chemotherapy agents, toceranib exhibited the most potent cytotoxic effects, hence, the decision to administer toceranib was based on its optimal efficacy. We closely monitored the patient’s response to toceranib and for any potential side effects. However, no toceranib-associated side effects, including anorexia, vomiting, diarrhea, gastrointestinal bleeding, hypertension, proteinuria, or the recently reported nephrotic syndrome (5, 16, 17), were noted. The owner reported that the patient remained in good condition and did not exhibit any pain response.

The suspected pulmonary metastatic lesions in our case were not confirmed through a histopathological examination due to the owner’s financial concerns. Thus, we could not definitively determine whether the pulmonary nodules were primary lung tumors or a product of the RCC metastasizing to the lungs. Nevertheless, pulmonary metastasis of the RCC was considered the most probable scenario, considering the high likelihood of these carcinomas metastasizing to the lungs. Furthermore, regression of the multiple nodules was evident on regular thoracic radiography. Additionally, after toceranib administration, the owner observed a significant improvement in the patient’s appetite and vitality. During regular follow-up, the number and size of multiple nodules suspected to be pulmonary metastases on thoracic radiography reduced, suggesting a positive response of the RCC to toceranib. Moreover, continuous monitoring for liver metastasis using abdominal ultrasonography did not reveal any metastasis to other organs within the abdominal cavity, despite the presence of blood vessels connecting the renal mass to the liver. Furthermore, no recurrence was observed despite the patient’s history of incomplete renal mass resection.

Toceranib is a multitarget TKI with inhibitory activity against various receptors, such as the vascular endothelial growth factor receptor, platelet-derived growth factor receptor, and Kit receptor (18). Thus, the drug may exhibit both an anti-angiogenic and antitumor potential (18). In our patient, the anti-angiogenic effects of toceranib might have contributed to the absence of additional metastasis within the abdominal cavity despite incomplete resection of the RCC. Moreover, the reduction in the sizes of the suspected metastatic pulmonary nodules could be attributed to these anti-angiogenic properties. Although TKIs have been widely utilized for the treatment of RCC in humans, their application in dogs with RCC is less common (19). For the treatment of mRCC in humans, several TKIs that specifically target the vascular endothelial growth factor signaling pathway have been approved as first-and second-line treatments, including sorafenib and sunitinib (20). The use of TKIs as targeted therapies has notably enhanced the survival rates in human patients with RCC (21). However, achieving a complete response to medical therapy in humans with mRCC is challenging. Three recognized clinical patterns are associated with the development of resistance in mRCC (22). A small percentage of patients show resistance to therapy from the beginning, whereas others experience initial tumor regression, followed by disease progression after a short period. However, some patients show an early positive response to treatment and maintain stable disease for an extended period (23).

Acute gastrointestinal disorder and drug resistance were speculated as the primary causes of our patient’s sudden death. Considering the reported cases of death from TKI-associated gastrointestinal ulceration or perforation (24–26), the potential of these complications as the acute causes of death cannot be ruled out, even though the patient exhibited no gastrointestinal adverse effects consistent with toceranib toxicity during regular check-up. Death could have also occurred due to reduced drug response caused by drug resistance, a phenomenon commonly observed in human mRCC cases. Despite the initial partial remission of the suspected metastatic pulmonary nodules during the first 2 months of treatment, no further improvement in the responsiveness was observed.

In veterinary medicine, the treatment options for highly malignant mRCC are challenging to determine owing to the rarity of these tumors and the limited number of previous studies. Consequently, clinicians encounter difficulties while selecting optimal chemotherapy for mRCC owing to the lack of sufficient evidence. Some reported carcinoma cases support the therapeutic effectiveness of toceranib as adjuvant chemotherapy with minimal side effects (6, 27–29). Regarding mRCC, only one recent case report has described the use of toceranib following nephrectomy with the aim of slowing the progression of metastatic lesions (30). This treatment led to a notable reduction in the size of lung masses, suspected to be metastatic lesions on computed tomography, and extended the patient’s survival time (30). Toceranib elicited a favorable response from the presumed metastatic pulmonary lesions in both the referenced case and in our case. Furthermore, in our case, taking into consideration the incomplete resection of the primary tumor, it effectively prevented any additional recurrences. Based on its high antitumor effect, indicated by both our anticancer drug response test as well as previous case reports, toceranib could be considered a treatment option for mRCC with favorable responsiveness.

To our knowledge, this is the first reported veterinary case of toceranib treatment in a canine with incompletely resected RCC and suspected pulmonary metastasis. The patient initially had a positive clinical outcome without any significant adverse effects. This case report provides valuable information on the clinical outcomes of toceranib therapy and the prognosis of incompletely resected RCC, a rare presentation in veterinary medicine. Further veterinary research on standard medical strategies and resistance to TKIs in canine RCC is warranted.

## Data availability statement

The original contributions presented in the study are included in the article/Supplementary material, further inquiries can be directed to the corresponding author.

## Ethics statement

The requirement of ethical approval was waived by Konkuk University Center for Research Ethics for the studies involving animals because this study is a case report, and only retrospective information about the patient was utilized. No separate experiments or sampling were conducted for the purpose of this report. The studies were conducted in accordance with the local legislation and institutional requirements. Written informed consent was obtained from the owners for the participation of their animals in this study. Written informed consent was obtained from the participant/patient(s) for the publication of this case report.

## Author contributions

D-EL: Writing – original draft. C-HN: Writing – review & editing. H-YY: Writing – review & editing. KB: Writing – review & editing. K-AY: Formal analysis, Writing – review & editing. J-HK: Writing – review & editing.
