# Supplementary material for: Case report: Adjuvant therapy with toceranib for an incompletely resected renal cell carcinoma with suspected pulmonary metastasis in a dog
Source: Front Vet Sci. 2023 Nov 13;10:1287185. doi: 10.3389/fvets.2023.1287185 (PMC10680256; doi:10.3389/fvets.2023.1287185)
Supplement: Supplementary file 1 [file Image_1.pdf]

Supplementary Material

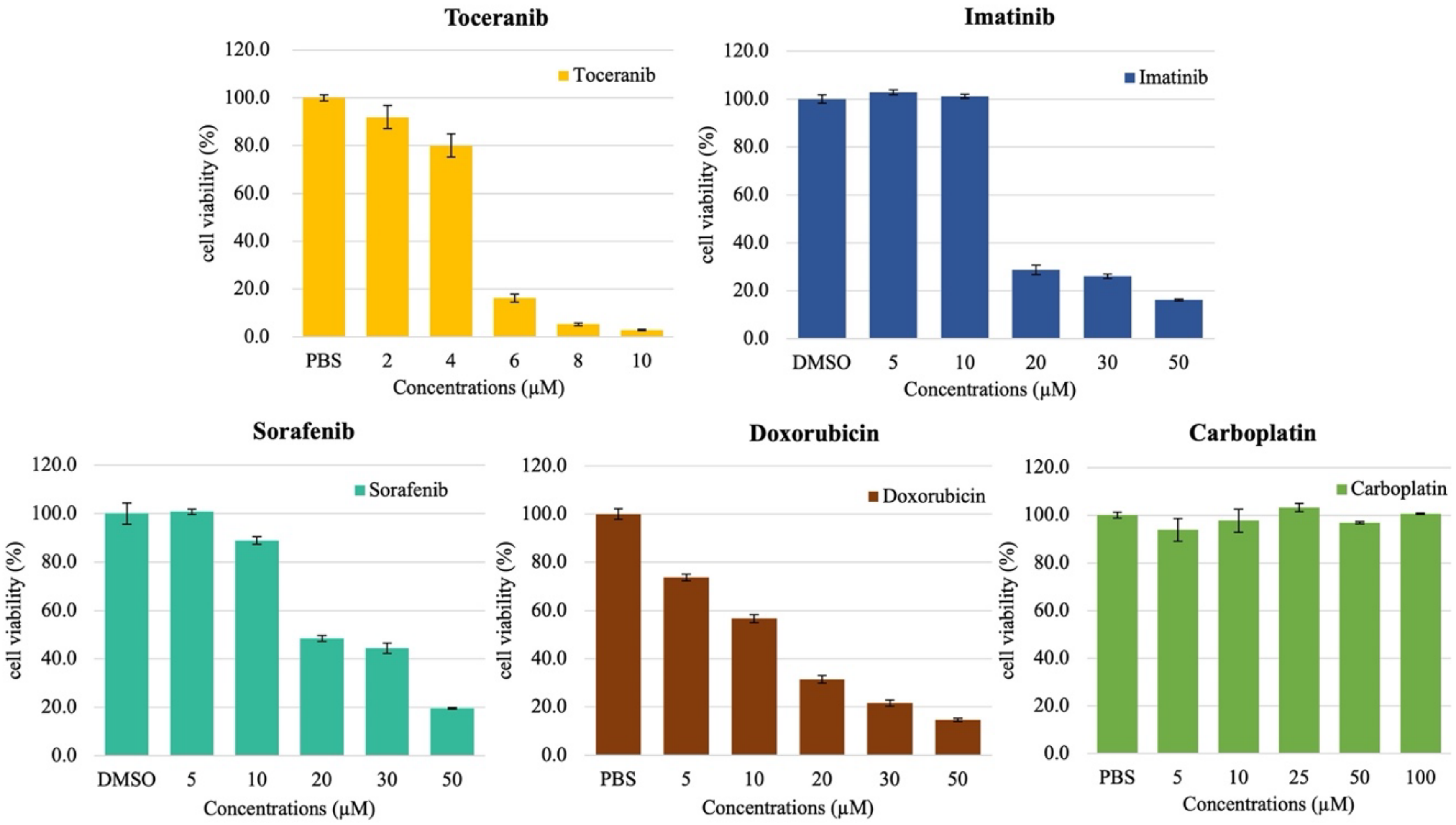

Supplementary Figure 1. *In vitro* anticancer drug response prediction test with toceranib, imatinib, sorafenib, doxorubicin, and carboplatin. Compared with the other chemotherapeutic agents, toceranib displayed a more potent antitumor activity at the lowest concentration of 6 µM. In contrast, carboplatin did not cause any cell death even at a high concentration of 100 µM. PBS, phosphate-buffered saline; DMSO, dimethyl sulfoxide.
